# Antiviral therapy in acute viral hepatitis B: why and when

**DOI:** 10.1186/1750-9378-4-2

**Published:** 2009-01-16

**Authors:** Giuseppe Morelli, Alessandro Perrella, Costanza Sbreglia, Pasquale Bellopede, Vincenzo Riccio, Oreste Perrella

**Affiliations:** 1 VII Department of Infectious Diseases and Immunology, "D. Cotugno" Hospital, Naples, Italy; 2 "Liver Unit," A. Cardarelli Hospital, Naples, Italy

## Abstract

Acute viral hepatitis B is cleared in more than 95% of patients, while the remainder ones may develop either chronic HBV infection or, rarely, fulminant hepatitis.

Therefore there are elderly patients with severe acute HBV hepatitis caractherized by high serum bilirubin levels >15 mmole/dl, international normalized ratio (INR) with value more than 1.6; these patients are caractherized by a severe outcome of HBV infection.

As known, outcome of infection and the pathogenesis of liver diseases are determined by viral and host factors, such as T reg lymphocytes.

T regs may be associated with a negative immune response such as an inhibition of gamma- IFN secretion.

The impact of viral load on antiviral T cell responses may play a critical role in thaese patients, influencing disease persistence and immune response.

Antiviral drugs could be useful in these patients determing a possible down -regulation of T regs.

## Letter to the editor

Dear Editor,

Acute viral hepatitis B is cleared in more than 95% of patients, while the remainder ones may develop either chronic HBV infection or, rarely, fulminant hepatitis. [1] The role of antivirals, such as lamivudine or entecavir, in acute HBV infection, has not been evaluated in controlled trials.

Lamivudine administration shows an antiviral efficacy in patients with HbeAg positive and HbeAg negative.

Among the patients with acute infection there are elderly subjects with severe course and severe outcome of HBV hepatitis.

A logical hypothesis for these patients is that a rapid decrease in the HBVDNA levels trough the use of antiviral agents could result in a less intense host response against HBV virus.

Today, increased knowledge of the virological and immunological events to HBV infection permits to define the mechanisms involved in viral clearance, persistence and disease severity.

Outcome of infection and the pathogenesis of liver disease are determined by viral and host factors. [1]

The impact of viral load on antiviral T-cell responses has been precisely characterized in animal models of viral infections: a sustained presence of viral antigens leads to virus specific T cell deletion [2].

In HBV infection the frequency and function of circulatory and intrahepatic HBV specific CD8 T cells is inversely proportional to the level of HBV-DNA. [3]

The immunological defects could be proportional to the level of HBV infection and inhibition of viral replication and through antiviral treatment it is possible to obtaine a partial restoration of HBV specific T cell immunity [4,5] which is inadeguate in elderly patients. [6]

Moreover recent studies have provided evidence that a population of specialized T cells are able to regulate the immune response.

These cells reside mainly within a minor population of CD4 cells that express the phenotype marker CD 25. [7]

They have been shown to suppress immunological responses against self and foreign antigens through suppressive cytokines.

It is possible that CD4+ CD25+ T cells are responsible for the weak HBV specific T cell response in HBV infection and may inhibit the expansion and function of HBV specific CD8 T cells precluding HBV clearance.

Some authors showed that the frequency of CD4+ CD25+ T cells positively correlate with HBVDNA load. [6]

This result suggests that an increased level of T regs may be associated with a negative immune response, leading to poor viral clearance.

Our hypothesis is that a decrease of HBV DNA load, determined by lamivudine treatment, determines a downregulation of T regs.

T regs could be able suppressing the population and gamma IFN production, mediated by HbsAg.

In summary, our findings suggest that it could be possible a marked increase in circulating Tregs in elderly patients with HBV infection.

Taken together these data, it is possible that antiviral drugs might be useful in a selected group of old patients in which HBV may cause a severe form of acute hepatitis, caused by a decline of immunity and by the frequent presence of other morbidities.

## Competing interests

The authors declare that they have no competing interests.

## Authors' contributions

GM performed the study and wrote the article.

AP and OP designed the study and reviewed the article.

CS, PB and VR analized the clinical data for the study.
