# Antiviral therapy in acute viral hepatitis B: why and when

**DOI:** 10.1186/1750-9378-4-5

**Published:** 2009-04-01

**Authors:** Giuseppe Morelli, Alessandro Perrella, Costanza Sbreglia, Pasquale Bellopede, Vincenzo Riccio, Antonio Monaco, Oreste Perrella

**Affiliations:** 1 VII Department of Infectious Diseases and Immunology, D Cotugno Hospital, Naples, Italy; 2 Liver Unit, A Cardarelli Hospital, Naples, Italy

## Abstract

Correction to Morelli G, Perrella A, Sbreglia C, Bellopede P, Riccio V, Perrella O: Antiviral therapy in acute viral hepatitis B: why and when. Infectious Agents and Cancer 2009, 4:2.

## Correction

After publication of this work [1], we noted that we inadvertently failed to include the complete list of all coauthors. The full list of authors has now been corrected here and the Authors' contributions and Competing interests section modified accordingly (see below).

## Competing interests

The authors declare that they have no competing interests.

## Authors' contributions

GM, AP were involved in preparation of data analysis, preparation of the manuscript and study coordination; CS, PB, VR, AM were involved in clinical investigation; OP was involved in idea writing of the manuscript, principal investigator and responsible for the study.
